# Effects of meal timing on changes in circulating epinephrine, norepinephrine, and acylated ghrelin concentrations: a pilot study

**DOI:** 10.1038/s41387-017-0010-0

**Published:** 2017-12-18

**Authors:** Simona Bo, Fabio Broglio, Fabio Settanni, Mirko Parasiliti Caprino, Alice Ianniello, Giulio Mengozzi, Antonella De Francesco, Maurizio Fadda, Debora Fedele, Alice Guggino, Ezio Ghigo, Mauro Maccario

**Affiliations:** 1 Department of Medical Sciences, University of Turin, Turin, Italy; 2 Clinical Biochemistry Laboratory, “Città della Salute e della Scienza” Hospital of Turin, Turin, Italy; 3 Unit of Clinical Nutrition, “Città della Salute e della Scienza” Hospital of Turin, Turin, Italy

## Abstract

**Background:**

Timing of food intake impacts on metabolic diseases. Few data are available about post-meal changes in epinephrine (E), norepinephrine (NE), and acylated ghrelin (AG) at different times of the day.

**Subjects and methods:**

This randomized cross-over trial investigated E/NE/AG concentrations after identical meals consumed at 0800 or 2000 hours in 20 healthy volunteers, by standardizing diet, exercise, duration of fast, and resting. Participants randomly received the test meal at 0800 or 2000 hours, and vice versa after 1 week. Blood samples were collected before and up to 180-min post-meal, every 30 min, with participants supine, motionless, but awake.

**Results:**

Median E levels increased at 30–60 min, then declined and rose again at 150 min; values at 60 min (19.0 vs. 15.0 ng/l, *p* = 0.03) and 180 min (25.0 vs. 11.0 ng/l, *p* < 0.001) were higher after the morning meals. NE rose at 30–60 min and then progressively declined; median values at 60 min (235.3 vs. 206.3 ng/l, *p* = 0.02) and 120 min (208.8 vs. 142.0 ng/l, *p* = 0.04) increased more after morning meals. AG progressively declined to increase again at 90 min after meal; median AG area-under-the-curve (AUC) values were lower at morning (7206.8 vs. 8828.3 pg/mL×h). AG-AUC was inversely associated with diet-induced thermogenesis (*β* = −121.6; 95% CI −201.0 to 42.2; *p* = 0.009 for each unit increase), while log NE-AUC was inversely associated with log-triglyceride AUC (*β* = −0.57; 95% CI −0.98 to 0.16; *p* = 0.015) in a multiple regression model, after multiple adjustments.

**Conclusions:**

In conclusion, E/NE concentrations were higher after the morning meal, while AG showed an opposite behavior. These data, although requiring confirmation in larger samples, suggest an adjunctive possible mechanism explaining the unfavorable effects of evening eating on metabolic risk

## Introduction

A growing evidence showed an association between meal timing and metabolic diseases in humans, suggesting that later food intake or more calories at evening have a negative impact on body weight, insulin resistance, hyperglycemia, hypertension, non-alcoholic fatty liver diseases, and the metabolic syndrome^1–3^.

The thermic effect of food, that is, the diet-induced thermogenesis (DIT), the increase in resting metabolic rate (RMR) after food ingestion, has been implicated in the development of obesity^4^.

DIT is lower in the evening^5, 6^, and we have recently found a 90.5 kcal increase in DIT after morning meals^5^. Nocturnal insulin resistance, slower evening gastric emptying, with increased carbohydrates absorption have been advocated as possible explanations^7, 8^. DIT consists of two components: the obligatory component, linked to nutrient metabolism, and the facultative one, likely mediated by the sympathetic nervous system^9^. The circadian rhythmicity of circulating norepinephrine (NE) and epinephrine (E) with increased values in the morning might explain the DIT daily variation^10^. Indeed, data about post-meal catecholamine concentrations are contrasting, since either a progressive E post-meal increase, unaltered values, a biphasic pattern with an initial rapid decline followed by a gradual rise^11, 12^ have been reported, while post-prandial NE values increase according to the meal nutrient composition, probably by higher spillover from sympathetic nerve endings^13^. An old, small study found a more pronounced increase in plasma catecholamines after lunch than after dinner^14^.

Finally, ghrelin, the hunger hormone released from the stomach and increasing food intake, oscillates in a circadian fashion and influences energy expenditure and thermogenesis, by suppression of brown fat thermogenesis^1, 15–17^; its primary action site has been reported to be the neuropeptide Y (NPY)/agouti-related peptide (AgRP) neurons in the hypothalamic arcuate nucleus^1^. NPY regulates the energy homeostasis, decreasing energy expenditure and inhibiting DIT in animal models^18^, and AgRP neurons have been reported to regulate energy expenditure, and thermogenesis in both brown and subcutaneous fat, via ghrelin receptor^16^. Therefore, the post-prandial variation in ghrelin concentrations might be involved in the regulation of DIT. Little is known about daily variations in morning vs. evening post-meal concentrations of this hormone and about its possible effect on DIT in humans.

The aim of the present pilot study was to evaluate catecholamines and acylated ghrelin (AG) concentrations after identical meals consumed in the morning (0800 hours) or in the evening (2000 hours), in conditions of standardized diet, physical activity level, duration of fast, and resting.

## Subjects and methods

The methods of this randomized cross-over trial have been previously reported^5^. Briefly, 20 healthy volunteers (10 males, 10 females) were enrolled among students/graduates attending the Department of Medical Sciences of Turin. Inclusion criteria were: 20–35 years, body mass index (BMI) 19–26 kg/m^2^, habitual moderate exercise level, smoking <10 cigarettes per day; exclusion criteria were: any acute or chronic diseases, menopause, any drug/supplement/specific diet, being shift/night workers, and incapacity to give written informed consent.

Participants randomly received a *standard meal* at 0800 hours and the week after the same meal at 2000 hours, or vice versa, to be consumed in 25–30 min. Before the meal (respectively at 2400 or 1200 hours), participants received the same standard meal (without protein supplementation) at their home, and were asked to spend the following 6 h in bed, refraining from drinking coffee, alcohol, or other beverages. The week preceding each test, the participants were instructed to maintain their usual diet and physical activity. The *standard meal* consisted of 100 g white bread, 100 g ham, 50 g cheese, 125 g yogurt, and 200 ml fruit juice; all foods were packed, and their composition is reported in Table 1. A 25 g protein supplement (Resource Instant Protein, Nestlè, Switzerland) was added to the *standard meal* the day of the test; the final nutritional composition (*standard meal* plus protein supplement) was 30% protein, 31% fat, and 39% carbohydrates; total kcal 1168. The adherence to the protocol by the participants was checked by phone calls.

**Table 1:** Nutritional composition of the standard meal without protein supplement

	**Quantity (g)**	**CHO (g)**	**Fat (g)**	**Protein (g)**	**Fiber (g)**	**Total kcal**
White bread	100	65.4	0.5	10.7	3.8	308.9
Pork cooked ham	100	0.1	18.1	26.3	0	268.7
Parmesan cheese	50	0	17.5	17.6	0	227.9
Peach yogurt^a^	125	19.0	5.0	5.0	0.2	141.0
Apricot fruit juice	200	29.0	0.2	0.9	0.2	121.5
Total		113.5	41.3	60.5	4.2	1068

*CHO* carbohydrates

^a^Whole milk

A 30-min basal calorimetric exam (Deltatrac II, DATEX, Division of Instruments Corp., Helsinki, Finland) was performed. At 0800 hours (or at 2000 hours), the participants consumed the meal, and then rested in a supine position for 90 min, followed by a second 60-min calorimetric evaluation. In order to obtain a better compliance to the experiment, the second calorimetric evaluation lasted 60 min (from 120 to 180 min from the beginning of the meal), since maintaining immobility while awaking was difficult for more than 1-h consecutive. Participants remained in a supine position but awake and motionless on a hospital bed for the whole period, except during the meal, when they were allowed to keep a sitting position and to void^5^. The exams were performed in a quiet room with a stable temperature of 23–25 °C, after warming-up the instrument for 30 min. After 5 min of initial calibration, gas samples were continuously analyzed by a paramagnetic and infrared gas chamber for sensing O_2_ and CO_2_, respectively^5^. Basal and after-meal RMR were calculated according to the formula of Weir; DIT was estimated as the difference between average after-meal RMR and the basal RMR^5^.

The Minnesota Leisure Time Physical Activity questionnaire and a 3-day food record, consisting of a detailed written food diary, was completed by all the participants^5^. Subjects were instructed to record everything they ate or drank during 2 consecutive week days and 1 weekend day.

Blood samples were collected before and every 30 min up to 180 min after the meal beginning.

Blood samples were immediately centrifuged and plasma aliquots were stored at −80 °C until the analysis. Serum glucose was measured by enzymatic colorimetric assay (Menarini Diagnostics, Florence, Italy), serum insulin by immunoradiometric assay (Beckman Coulter, Immunotech, Prague, Czech Republic; intra-assay coefficients of variation ≤3.99% and inter-assay coefficients of variation ≤4.8%). Free fatty acid (FFA) concentrations were measured by a fluorometric assay (Sigma-Aldrich, St. Louis, MO, USA), and plasma triglycerides by enzymatic colorimetric method (Hitachi, Roche Diagnostics, Mannheim, Germany).

Plasma E/NE were measured by chromatographic determination on isocratic high-performance liquid chromatography (HPLC) system with electrochemical detector fixed with a potential of 400–500 mV (Chromsystems Instruments & Chemicals GmbH, Gräfelfing, Germany). Briefly, blood samples were mixed with 20 µl stabilization solution, and plasma samples were obtained by centrifugation. Plasma and Internal Standard (IS) were mixed and applied to a sample clean-up cartridge for a solid-phase extraction. Cartridges were mixed, centrifuged, and washed. Amount (20–50 μl) of eluates were injected into the HPLC system, and the retention times of E, NE, and IS were, respectively, 5.8, 7.0, and 10.5 min. For both E/NE, the limit of quantification was 18–43 ng/l, and intra-assay and inter-assay coefficients of variation were 3.8–6.0%. AG concentrations were assessed by ELISA (enzyme-linked immunosorbent assay) based on double-antibody sandwich technique (DRG International Inc., Springfield Township, NJ, USA) (limit of quantification 4 ng/l; intra- and inter-assay coefficients of variation 7.0–11.2%).

Due to the nature of the intervention, blinding was not feasible. The laboratory personnel who performed the biochemical analyses was blinded to the group assignment.

The number of 20 subjects was required to test a 0.66 DIT effect size with a power = 80% and a two-tailed *α*-value = 0.05.

The study was approved by the local ethics committee; all the procedures conformed to the Declaration of Helsinki principles; all the participants provided written informed consent to participate.

Variables were presented as mean and standard deviation (SD) or, when not-normally distributed, as median (interquartile range). The Student’s t-test for paired data or the Wilcoxon's matched paired test (not-normally distributed variables) were used to investigate within-subject differences of the variables after the morning and evening meals.

The net AUC values were calculated by the trapezoidal method^5^.

The associations between AUC values of hormones with DIT and metabolic variables were evaluated by a multiple regression model, using log-transformed variables, as appropriate, and adjusting for age, sex, and BMI.

## Results

The participants’ mean anthropometric characteristics and laboratory values before the *standard meal* are reported in Table 2. Participants were moderately active, and usually consumed a high-fat (39.9% total kcal) low-fiber (11.9 g/day) diet. All the participants were non-smokers.

**Table 2:** Characteristics of the participants and laboratory variables at time 0 (before the standard meal)

Males/females	10/10
Age (years)	27.6 ± 3.4
Weight (kg)	67.3 ± 12.5
BMI (kg/m^2^)	23.4 ± 3.2
Glucose (mg/dl)	77.4 ± 12.5
Insulin (µU/ml)	3.5 (3.4)
Triglycerides (mg/dl)	62.0 ± 21.0
Free fatty acids (mmol/l)	0.64 (0.54)

Data are presented as mean ± SD or median (interquartile range) for not-normally distributed variables

Median post-meal E levels increased at 30–60 min, then declined and rose again at 150 min (Table 3). The values at 60 and 180 min were significantly higher after the morning meal. NE rose at 30–60 min and then progressively declined; values at 60 and 120 min were higher after morning meals.

**Table 3:** Epinephrine, norepinephrine, and acylated ghrelin concentrations by timing of the meal

	**Morning meal**	**Evening meal**	P*
Epinephrine 0 (ng/l)	10.0 (7.5)	10.0 (5.0)	0.27
Epinephrine 30 (ng/l)	20.0 (7.0)	14.0 (17.0)	0.43
Epinephrine 60 (ng/l)	19.0 (21.0)	15.0 (11.0)	0.03
Epinephrine 90 (ng/l)	10.0 (13.0)	14.0 (9.0)	0.83
Epinephrine 120 (ng/l)	10.0 (33.0)	12.5 (10.0)	0.09
Epinephrine 150 (ng/l)	17.5 (13.5)	15.0 (10.0)	0.09
Epinephrine 180 (ng/l)	25.0 (5.0)	11.0 (10.0)	<0.001
AUC Epinephrine (ng/l×h)	3142.5 (3225.0)	3045.0 (1650.0)	0.07
Norepinephrine 0 (ng/l)	137.7 (93.0)	165.0 (121.6)	0.30
Norepinephrine 30 (ng/l)	214.0 (101.5)	247.0 (208.8)	0.12
Norepinephrine 60 (ng/l)	235.3 (115.0)	206.3 (200.1)	0.02
Norepinephrine 90 (ng/l)	198.1 (120.0)	182.5 (129.6)	0.18
Norepinephrine 120 (ng/l)	208.0 (88.5)	142.0 (90.3)	0.04
Norepinephrine 150 (ng/l)	164.0 (97.0)	130.0 (134.2)	0.71
Norepinephrine 180 (ng/l)	157.5 (35.8)	132.4 (124.0)	0.46
AUC Norepinephrine (ng/l×h)	36 390.0 (14,760.8)	33 219.0 (23 928.0)	0.15
Acylated ghrelin 0 (ng/l)	64.2 (21.0)	67.6 (37.1)	0.82
Acylated ghrelin 30 (ng/l)	41.7 (28.7)	40.6 (32.1)	0.82
Acylated ghrelin 60 (ng/l)	23.9 (37.1)	30.2 (38.4)	0.82
Acylated ghrelin 90 (ng/l)	29.7 (24.7)	34.1 (33.7)	0.50
Acylated ghrelin 120 (ng/l)	38.9 (24.4)	45.8 (41.6)	0.12
Acylated ghrelin 150 (ng/l)	42.9 (21.6)	53.6 (54.4)	0.04
Acylated ghrelin 180 (ng/l)	49.2 (22.9)	58.4 (61.9)	0.02
AUC acylated ghrelin (ng/l×h)	7206.8 (4414.7)	8828.3 (7136.1)	0.04

Values are reported as median (interquartile range)

*AUC* net area under the curve calculated by the trapezoidal method

*p value was obtained by Wilcoxon's matched paired test

AG progressively declined after meal to increase thereafter at 90 min. The increment was more pronounced after the evening meals.

**Fig. 1:**
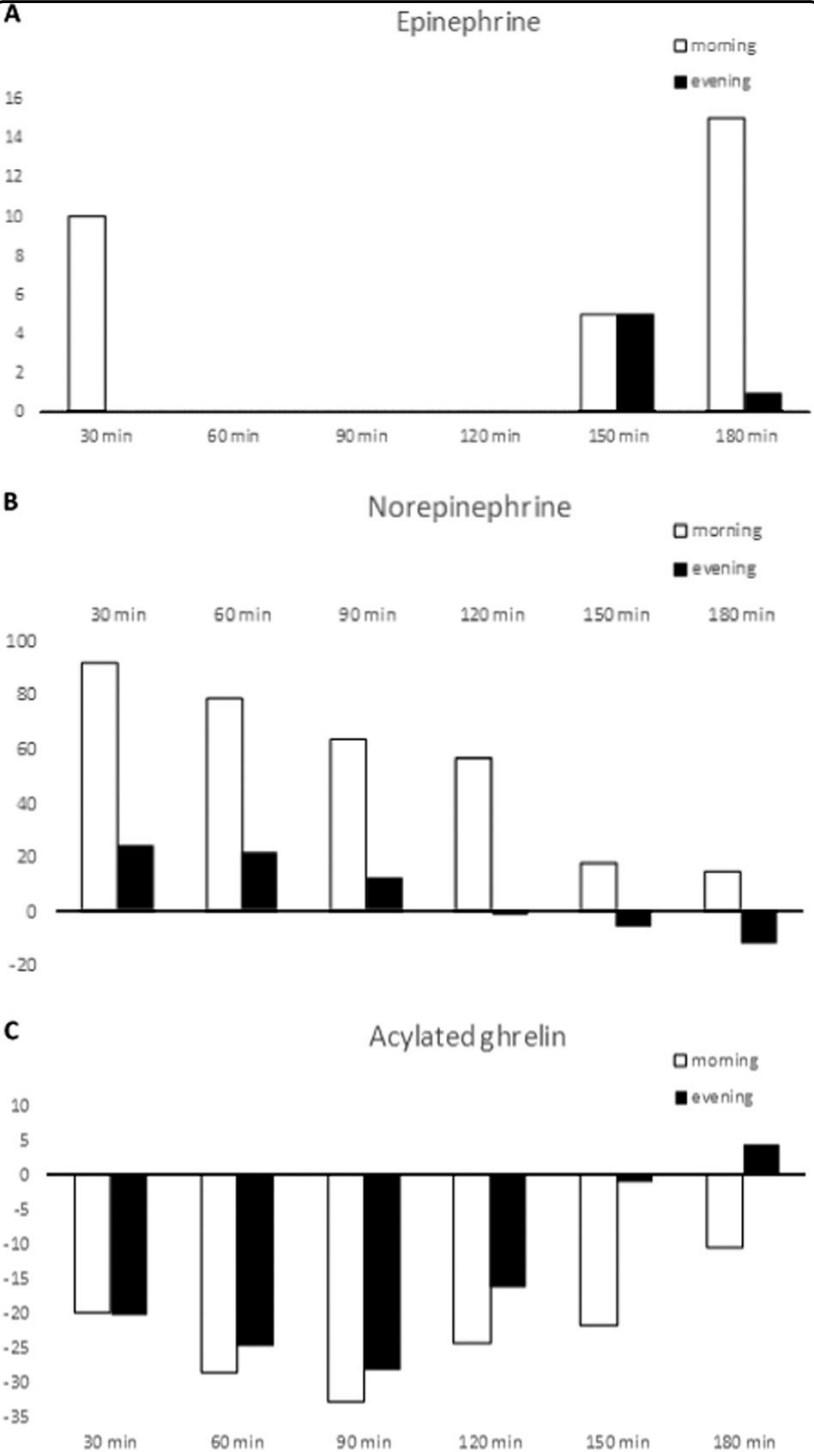
**Median variation of epinephrine, norepinephrine, and acylated ghrelin at the different time points by timing of the meal.** Median variation of epinephrine (**a**), norepinephrine (**b**), and acylated ghrelin (**c**) from time 0 (before meal) at the different time points

The median values of the difference between the time point measurements and the before-meal measurements after the morning or evening meals are reported in Fig. 1. Changes in E levels were significantly different at 120 min (*p* = 0.02) and at 180 min (*p* = 0.002), while changes in NE differed at 30 min (*p* = 0.008), 60 min (*p* = 0.03), 90 min (*p* = 0.04), 120 min (*p* = 0.04), and 180 min (*p* = 0.048).

Mean morning DIT values were significantly higher than evening DIT values (327.5 ± 103.7 vs. 237.0 ± 89.5 kcal, *p* = 0.003). Evening log AG-AUC was inversely associated to DIT in a multiple regression model, after multiple adjustments (*β* = −121.6; 95% CI −201.0 to 42.2; *p* = 0.009 for each unit increase). No other significant associations with DIT values were found.

Mean AUCs for glucose, insulin, triglyceride, and FFA blood concentrations were, respectively 15 383.2 ± 2045.3 mg/dl × h, 6968.9 ± 2788.7 µU/mL × h, 11 721.0 ± 4118.3 mg/dl × h, 38.7 ± 16.8 mmol/L × h after the morning meal, and 17 183.3 ± 2290.4 mg/dl × h, 8597.8 ± 4231.1 µU/mL × h, 12 363.0 ± 2767.9 mg/dl × h, and 54.7 ± 30.3 mmol/L × h after the evening meal, with significantly higher evening values for glucose AUCs (*p* < 0.001), log-insulin AUCs (*p* = 0.007), and FFA AUCs (*p* = 0.04).

We found a significant inverse association between log NE-AUC and log-triglyceride AUC in a multiple regression model (*β* = −0.57; 95% CI −0.98 to 0.16; *p* = 0.015).

## Discussion

E/NE concentrations tended to be higher after the morning rather than the evening meals, while AG showed an opposite behavior. Particularly, evening AG-AUC was inversely correlated to evening DIT.

### Epinephrine and norepinephrine

Feeding increases sympathetic activity; in our pilot study, post-meal E concentrations showed a biphasic pattern, in line with the literature^12^, while post-meal NE values initially increased and then gradually declined^13^. Indeed, NE is released by postganglionic sympathetic nerve terminals, while E is secreted almost exclusively by the adrenal medulla. A circadian rhythmicity of circulating E/NE has been found, with increased values of both compounds in the morning and lower nocturnal levels, but specific major differences in E/NE release patterns were reported, suggesting separate factors controlling their circulating levels^10^. Our data confirmed a dissociation between post-meal adreno-medullary and sympathetic activities.

Overall, E/NE concentrations in our participants were higher after the morning meal. Accordingly, catecholamines showed a more pronounced rise after lunch at 1200 hours than after dinner at 1700 hours in a small study^14^.

The morning/evening sympathetic differences might explain the increased morning DIT we have found in our participants^5^. A direct relation between calorie ingestion and sympathetic activity has been established^9^. However, human studies examining adrenergic activity in obesity have given conflicting results^13, 19^. Lower level of sympathetic activity and NE responsiveness were reported after overfeeding in obese when compared to lean individuals, thus reducing the energy expenditure induced by overfeeding, with subsequent weight gain^19^ However, the reduced thermic response to feeding seen in obese subjects cannot be directly explained by the diminished sympathetic nervous system activity as reflected by NE plasma levels^20^. Accordingly, we did not find a significant association between DIT and E/NE-AUCs, suggesting a marginal role for these hormones, in line with the reported small sympathetic contribution to diet-induced energy expenditure in normal subjects, and the absence of a direct relationship between post-prandial thermogenesis and sympathetic activity^13^. Furthermore, morning/evening differences in DIT have been related primarily to the endogenous circadian system and not to the behavioral cycle or the sympathetic activation^6^. The evening decrement in insulin sensitivity with a decreased thermic effect of glucose due to the diminished insulin-mediated glucose uptake and metabolism by skeletal muscle might result in diminished glucose-induced thermogenesis, too^5^.

The relationships between catecholamines and metabolic variables are controversial. We found an inverse association between log NE-AUC and log-triglyceride AUC. Old studies reported acute stress-mediated reduction of plasma triglyceride concentrations via the sympathetic nervous system^21^ and suppression of the secretion of triglycerides and apoprotein B by NE from rat liver^22^. Catecholamines stimulate both the α- and β-adrenergic systems which induce the stimulation of adenylate cyclase and protein kinase; thereafter, increased adipose triglyceride lipase and hormone-sensitive lipase promote triglyceride hydrolysis in the adipose tissue^23^.

Catecholamines have uniformly been reported to increase plasma FFA levels by enhancing lipolysis during fasting^24^. In our post-meal tests, FFA concentrations progressively declined after the meal, as expected, with FFA AUCs being higher after the evening meal ^5^. We failed to find any significant association between E/NE and FFA values; the increased evening insulin resistance might be the major determinant of the higher increase in evening FFA-AUC, even if our data do not allow to establish a causal relationship^5^. Accordingly, in healthy subjects, the suppression of the lipolytic hormone-sensitive lipase has been shown to be more marked when induced by endogenous insulin secretion after glucose ingestion rather than by stress-induced endogenous catecholamines^25^. The interference of the meal, the peculiarity of the test conditions, and the relatively short duration of the observation (up to 180-min post-meal) might, therefore, have contributed to our results. Further studies on larger samples, including other factors related to the sympathetic nervous system are required to obtain definitive results.

### Acylated ghrelin

AG levels surge before mealtime, stimulating eating, with diurnal fluctuation and a trough at night^26^. We found higher evening AG levels, even if morning/evening changes from baseline at the different time points did not differ significantly. A suppression and subsequent rebound of AG levels by food intake, independently from the day- time, and an inhibitory effect of sleep have been reported^27^. Insulin release has been implicated in the post-meal ghrelin inhibition^27^; furthermore, ghrelin release in mice and humans timed to previous mealtimes persisted for days of fasting, suggesting that rhythm phase is controlled by food availability time^26^. It could therefore be hypothesized that both the evening insulin resistance and the tendency to consume bigger evening meals justified the higher AG levels we found in the evening.

The demonstration of a negative association between AG-AUC and evening DIT is intriguing. Both these conditions could result in weight gain, and their association suggests a potential additive effect. Ghrelin has an excitatory effect on AgRP/NPY neurons, and NPY plays a critical role in the regulation of energy homeostasis, inducing decrease in energy expenditure, inhibiting DIT, and promoting weight and fat gain^18^. In brown adipocytes, ghrelin decreases the expression of thermogenic regulators, whereas the ablation of the GHS‐R1a (growth hormone secretagogue receptor) ghrelin receptor (which selectively binds only the acylated form of ghrelin) activates thermogenic signaling, thus suggesting that via the activation of this receptor, AG regulates thermogenesis^15^. Similarly, in AgRP neuron-specific GHS-R knockout mice, diet-induced obesity was mitigated by thermogenesis activation^16^.

Therefore, the post-prandial decrease in ghrelin may be involved in the regulation of DIT. In a small pilot human study, a lower DIT-induced meal determined a higher ghrelin increase, even if no significant associations were found^28^. In healthy women with a broad range of BMI, a significant inverse correlation between post-meal RMR and baseline ghrelin blood concentrations was found, independent of individual differences in fat-free mass and fat mass^29^. These data confirmed a negative regulation role for ghrelin in energy homeostasis; the increased evening AG concentrations might be one of the mechanisms explaining the unfavorable effects of big evening meals. Indeed, the regulation of ghrelin release is a complex process that is tightly controlled by the sympathetic nervous system and the gastrointestinal tract, involving hormonal stimuli not necessarily implicated in energy balance regulation.

Reciprocal relationships among ghrelin and blood insulin, glucose concentrations, and BMI have been reported^17^. We failed to find such correlations, however, in this pilot study, we have studied an acute response after a standard meal in healthy individuals, and the effects of ghrelin on glucose homeostasis and metabolism seem to be associated with the energy state and nutrition, depending on the environmental conditions of the experiments^15, 17, 30^.

### Limitations

In this pilot study small sample size leading to the possibility of type II errors, its short-term duration, the lack of measurements of other factors related to the sympathetic nervous system, and of gastrointestinal/pancreatic hormones were all potential limitations of this trial. Furthermore, we did not measure total and des-acyl ghrelin; however, the main effects of ghrelin on food intake are exerted by the acylated molecule through its interaction with the GHS-R1a, and the des-acyl compound that does not activate this receptor has been reported to play no thermogenic effect^15^.

Finally, our results were obtained after a high-protein meal. It is well known that DIT is influenced by the energy intake and the macronutrient composition of the meal; in particular, high-protein or high-carbohydrate meals increase DIT more than high-fat meals^31^. Increases in catecholamine concentrations were found especially with high-carbohydrate meals^13, 32^, while ghrelin reduction is proportional to the caloric and macronutrient composition of meals, with fats being the least effective ghrelin suppressors^17, 33^.

In all, although the study design, the carefully controlled experimental conditions, and the sensitive assays employed can be considered as the strengths of this trial, the aforementioned limits suggest to considering our results as preliminary and specific to our experimental context. Further confirmatory studies with larger sample would be needed to obtain conclusive results.

## Conclusions

In conclusion, the post-meal lower E/NE and the increased AG concentrations, inversely associated to DIT values, support the unfavorable effects of evening eating. These data add further evidence about the importance of meal timing on weight gain risk, although further investigation in larger samples are required for a definitive confirmation.
